# Striving or surviving? Influence of bullying on psychological well-being and coping approaches among undergraduate dental students

**DOI:** 10.12669/pjms.42.8.15956

**Published:** 2026-08

**Authors:** Preh Ayub, Junaid Sarfraz, Saleem Raza, Yousif Ali Shah

**Affiliations:** 1 Preh Ayub, BDS, MSPH, MHPE, (Dip.O.Med), SMBB Medical University, Larkana, Sindh, Pakistan, Health Services Academy, Islamabad, Pakistan; 2 Junaid Sarfraz Khan, MBBS, FCPS, FRCS, Ph.D. Health Services Academy, Islamabad, Pakistan; 3 Saleem Raza Khuhawar, BDS, M.Phil., (Ph.D.), SMBB Medical University, Larkana, Sindh, Pakistan; 4 Syed Yousif Ali Shah, BDS, MSC, Ph.D. SMBB Medical University, Larkana, Sindh, Pakistan

**Keywords:** Anxiety, Bullying, Coping Strategies, Depression, Dental Students, Mental Health

## Abstract

**Objectives:**

To assess the frequency of bullying and its association with depression, anxiety, stress, and coping strategies among undergraduate dental students.

**Methodology:**

A cross-sectional study was conducted among 124 dental undergraduates at Bibi Aseefa Dental College, Larkana, Pakistan (August 2025–January 2026). Bullying exposure was assessed using an adapted 15-item Negative Acts Questionnaire–Revised (NAQ-R), while psychological distress was measured using the 21 items Depression, Anxiety, and Stress Scale (DASS-21), Coping strategies were evaluated using the 24 items Brief-COPE inventory and were categorized as adaptive and maladaptive. Data were analyzed using descriptive statistics, Chi-square tests, and Spearman’s correlation, with a p-value < 0.05 considered statistically significant.

**Results:**

Most students reported high bullying exposure (74.2%). Moderate to extremely severe depression was observed in 50.8%, severe to extremely severe anxiety in 54.0%, and moderate to severe stress in 29.9% of participants. Bullying exposure was significantly associated with depression (χ² = 18.2, p = 0.020), stress (χ² = 16.6, p = 0.035), and anxiety (χ² = 29.2, p < 0.001). Spearman’s correlation revealed that bullying was positively correlated with anxiety (ρ = 0.427, p < 0.001) and maladaptive coping (ρ = 0.403, p < 0.001), while maladaptive coping strongly correlated with anxiety (ρ = 0.648, p < 0.001). Adaptive coping showed weaker positive correlations with anxiety, suggesting increased coping efforts in response to distress. Multivariable analysis identified high bullying exposure (OR ≈6.0, p<0.001) and maladaptive coping (OR ≈ 1.18, p<0.001) as significant independent predictors of moderate to severe anxiety.

**Conclusion:**

Bullying is highly prevalent among dental students and strongly associated with anxiety, depression, and maladaptive coping. Targeted interventions, including bullying prevention, mental health support, and resilience training, are essential to safeguard student well-being.

## INTRODUCTION

Dental education is widely acknowledged as one of the most challenging academic paths, requiring a blend of advanced cognitive abilities and precise psychomotor skills. However, the traditional peer relationship commonly present in clinical settings may unintentionally lead to a culture of mistreatment. Medical and dental students often endure intense psychological pressure due to tough coursework and cutthroat environments that require significant personal and social sacrifices. Students are frequently exposed to stressful situations where the boundary between rigorous training and psychological aggression becomes blurred.^1^ Bullying is a widespread phenomenon that occurs across diverse settings. It involves intentional or recurring actions, or intermittent behaviors that are offensive, abusive, intimidating, or degrading toward an individual. Such conduct may include mistreatment, unfair penalties, or physical misconduct, making people feel angry, humiliated, scared, or vulnerable. Previous studies have reported shockingly high rates of bullying in dental colleges, with 137 (91.3%) dental students reporting experiences of various forms of bullying within the past twelve months.^2^

The impact of bullying extends beyond academic performance. Long term exposure to bullying is a major contributor to mental health issues, with research consistently demonstrating a clear link between mistreatment and the triad of depression, anxiety, and stress.^3^ Tough academic atmosphere may also lead to burnout and reduced empathy toward patients.^4^ Students are not merely passive recipients of these experiences; they employ a range of coping mechanisms to manage such challenges. The high academic demands and hierarchical structures inherent in dental education may increase students’ vulnerability to bullying and adversely affect their mental health.^5^ Despite growing awareness of mental health challenges in dental education, research in the local context remains limited.^6^ Dental College in Larkana, Pakistan, is a key regional institution; however, no previous studies have examined the mental health impact of bullying or the coping strategies adopted by its students. Therefore, this study was undertaken to assess the prevalence of bullying and its association with psychological distress and coping mechanisms among dental students at this institution.

## METHODOLOGY

The cross-sectional study was carried out in BADC, Larkana (August 2025 -January 2026). A total population sampling method was employed and the entire population of the enrolled undergraduate dental students were invited to take part. The number of students who gave informed consent was 160 out of 185 (response rate: 86.5%). Fifteen were not counted because they were pilot-tested and 21 were not counted because of missing data. Therefore, 124 completed questionnaires have been used in the final analysis, which produced effective response rate of 77.5% (124/160). Both genders participated and completed a self-administered online questionnaire. Bullying exposure was measured using an adapted 15 items Negative Acts Questionnaire–Revised (NAQ-R), where eight out of 23 items in the original item scale were dropped.

Removed items were mostly associated with professional retaliation (e.g., being assigned to work with uncontrollable workload), behavior that are specific to the role of a manager (e.g., having your opinions neglected by your supervisor), and negative behaviors at the organizational level (e.g., being assigned tasks with unrealistic deadlines), which presuppose an employer-employee relationship which is not relevant to undergraduate dental students A panel of three experts in dental education and public health reassessed content validity of the adapted 15 items NAQ-R, and confirmed that all retained items were relevant. The modified instrument was then pilot-tested on 15 dental students (not included in the final sample), which led to the slight changes in wording but no additional item eliminations. The overall 15 items NAQ-R adapted was used to classify bullying exposure scores into three levels low (scores 15-22), moderate (scores 23-30) and high (scores 31-75). This is the classification approach that is consistent with prior NAQ-R validation studies.

Depression, anxiety, and stress were assessed using the 21 items Depression Anxiety Stress Scale (DASS-21) (Lovibond & Lovibond, 1995). Categories of severity were determined on the basis of the standard DASS-21 cut-off scores: Depression (Normal 0-9, Mild 10-13, Moderate 14-20, Severe 21-27, Extremely Severe 28+); Anxiety (Normal 0-7, Mild 8-9, Moderate 10-14, Severe 15-19, Extremely Severe 20+); Stress (Normal 0-14, Mild 15-18, Moderate 19-25, Severe 26-33, Extremely Severe 34+). Coping strategies were evaluated using an adapted 24-item Brief-COPE inventory(Carver, 1997), with coping strategies categorized into adaptive and maladaptive subscales. These strategies were categorized into subscales: adaptive coping (planning, acceptance, active coping, use of emotional and instrumental support, positive reframing) and maladaptive coping (self-blame, denial, venting, substance use, behavioral disengagement). All instruments demonstrated good reliability, with Cronbach’s alpha values of 0.876 (NAQ-R), 0.934 (DASS-21), and 0.884 (Brief-COPE). Cronbach’s alpha values ≥ 0.7 indicate acceptable internal consistency, demonstrating reliability of the instruments for this study population. All the participants gave their written informed consent. The consent form contained a description of the study purpose, voluntary participation, the right to withdraw without penalty and anonymity. Questionnaires were coded and the information stored in a computer that is secured with a password and only accessible to the principal investigator, only aggregated data will be reported and electronic data destroyed at the end of five years.

### Ethical Approval:

Institutional Review Committee gave ethical approval (Approval No: F: No HSA/Rector/002-26, Date: August 6, 2025).

### Statistical analysis:

Data were analyzed using Jamovi version 2.6.45.0. Descriptive statistics summarized participant characteristics and scale scores. Associations between demographic factors, bullying exposure and depression, anxiety, and stress were examined using Chi-square tests, while Spearman’s rank correlation assessed relationships between bullying, coping strategies, and anxiety. Additionally, multivariate logistic regression analysis was performed to identify independent predictors of moderate to severe anxiety (dichotomized as normal/mild vs. moderate/severe/extremely severe), adjusting for potential confounders including age, gender, study year, residence, and history of examination failure. A significance level of p < 0.05 was considered statistically significant.

## RESULTS

The study examined the demographic characteristics and key study variables among the participants. The demographic profile of the respondents, including age, gender, academic year, and history of examination failure, is presented in Table-I.

**Table-I:** Demographic characteristics of undergraduate dental students (n = 124).

Variables	Category	n	%
Gender	Female	66	53.2
	Male	58	46.8
Age	18-20	38	30.6
	21-22	64	51.6
	23-25	22	17.7
Residence	Hostel	97	78.2
	Home	27	21.8
Study Year	1st	33	26.6
	2nd	27	21.8
	3rd	32	25.8
	4th	32	25.8
History of Exam Failure	Yes	21	16.9
	No	103	83.1

### Demographic Profile:

Severity Distribution of Depression, Anxiety, and Stress among Undergraduate Dental Students (Table-I):

Values are presented as frequency (percentage). Severity categories are based on DASS-21 scoring guidelines. Table-II

**Table-II:** Distribution of Depression, Anxiety, And Stress (n = 124).

Severity Level	Depression		Anxiety		Stress	
	n	%	n	%	n	%
Normal	42	33.9	21	16.9	72	58.1
Mild	19	15.3	11	8.9	15	12.1
Moderate	38	30.6	25	20.2	21	16.9
Severe	11	8.9	22	17.7	11	8.9
Extremely Severe	14	11.3	45	36.3	5	4.0
Total	124	100	124	100	124	100

### Association Between Bullying Exposure and Mental Health Outcomes:

The most striking finding was made on the anxiety dimension: of the students who reported high levels of bullying exposure, 44.6% had extremely severe anxiety levels compared to 15.4% in the low-bullying group (29.2, df = 8, p < 0.001). This is a almost three times greater percentage of extremely severe anxiety in the high-bullying group, which illustrates the devastating psychological effect of bullying. Table-III

**Table-III:** Association between bullying exposure and depression, anxiety and stress among undergraduate dental students (n = 124).

Bullying	Normal	Mild	Moderate	Severe	Extremely Severe	Total
	Depression Severity					
Low	9(69.2%)	2(15.4%)	1(7.7%)	1(7.7%)	0(0%)	13
Moderate	9(47.4%)	5(26.3%)	4(21.1%)	1(5.3%)	0(0%)	19
High	24(26.1%)	12(13.0%)	33(35.9%)	9(9.8%)	14(15.2%)	92
Total	42	19	38	11	14	124
χ² = 18.2, df = 8, p = 0.020						
***(B) Stress Severity***						
Low	12(92.3%)	1(7.7%)	0(0%)	0(0%)	0(0%)	13
Moderate	14(73.7%)	4(21.1%)	1(5.3%)	0(0%)	0(0%)	19
High	46(50.0%)	10(10.9%)	20(21.7%)	5(12.0%)	5(5.4%)	92
Total	72	15	21	11	5	124
χ² = 16.6, df = 8, p = 0.035						
***(C) Anxiety Severity***						
Low	7(53.8%)	1(7.7%)	2(15.4%)	1(7.7%)	2(15.4%)	13
Moderate	7(36.8%)	1(5.3%)	6(31.6%)	3(15.8%)	2(10.5%)	19
High	7(7.6%)	9(9.8%)	17(18.5%)	18(19.6%)	41(44.6%)	92
Total	21	11	25	22	45	124

χ² = 29.2, df = 8, p < 0.001.

### Association of Demographic Variables with Mental Health Outcomes:

Association between participants’ demographic characteristics and mental health outcomes were examined. Gender, residence, and history of examination failure were not significantly associated with anxiety, stress, depression, or bullying exposure. Similarly, gender differences in coping strategies were not statistically significant. In contrast, a statistically significant association was observed between year of study and anxiety severity (χ² = 41.3, df = 12, p < 0.001), with higher anxiety levels observed among senior-year students.

### Descriptive statistics of Brief COPE inventory subscales (Table-IV):

**Table-IV:** Spearman’s correlation between bullying exposure, coping strategies, and anxiety severity (n = 124).

	Adaptive Coping	Maladaptive Coping	Anxiety	Bullying
Adaptive Coping	-			
Maladaptive Coping	0.633**	-		
Anxiety	0.363**	0.648**	-	
Bullying	0.262*	0.403**	0.427**	-

Values indicates Spearman’s correlation coefficients (ρ).

* p = 0.003

** p < 0.001.

Descriptive analysis of coping strategies showed that planning had the highest mean score (mean = 5.50, SD = 2.17), followed by active coping (mean = 5.06, SD = 1.81) and positive reframing (mean = 5.01, SD = 1.75). Acceptance (mean = 4.73, SD = 1.55), self-distraction (mean = 4.72, SD = 1.53), denial (mean = 4.88, SD = 1.71), and self-blame (mean = 4.40, SD = 1.74) showed moderate utilization. Venting (mean = 4.22, SD = 1.62), instrumental support (mean = 4.17, SD = 1.57), and emotional support (mean = 4.12, SD = 1.72) were comparatively lower. Substance use (mean = 3.35, SD = 1.55) and behavioral disengagement (mean = 3.83, SD = 1.64) had the lowest mean scores among the coping strategies assessed.

### Multivariate logistic regression analysis (Table-V):

**Table-V:** Multivariate logistic regression analysis for Moderate to Severe Anxiety.

Predictor	Adjusted OR	95%CI	p-value
Bullying (High vs Low)	~4.2	1.5-11.8	0.006
Bullying (Moderate vs Low)	~1.3	0.4-3.9	0.62
Maladaptive Coping	~1.18 (per unit)	1.08-1.30	<0.001
Adaptive Coping	~0.096	0.89-1.04	0.28
Gender (Male vs Female)	~1.0	0.5-2.2	0.90
Year	NS	-	>0.05
Residence	NS	-	>0.05
Exam Failure	NS	-	>0.05

**Fig.1:**
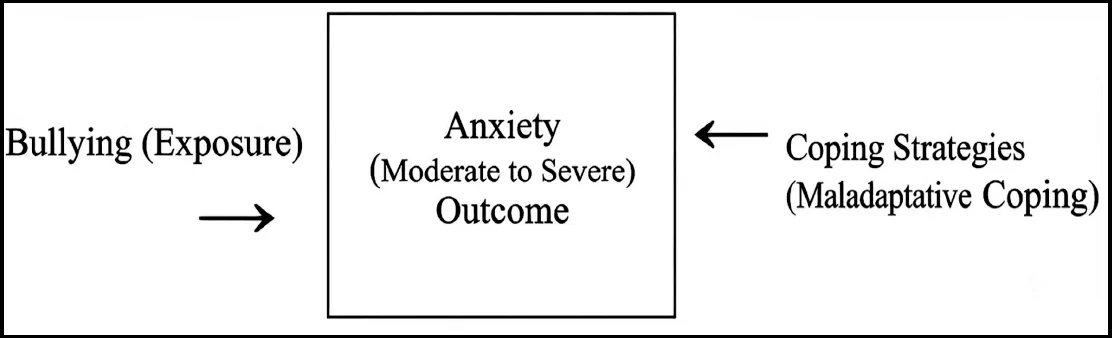
Conceptual framework illustrating the relationship between Bullying Exposure, Maladaptive Coping Strategies, and Anxiety Outcomes.

Performed to identify independent predictors of moderate to severe anxiety among dental students. After adjusting for gender, academic year, residence, and examination failure, high bullying exposure emerged as a strong independent predictor of anxiety (Adjusted OR ≈ 6.0, p < 0.001). Students experiencing high levels of bullying were approximately six times more likely to report moderate to severe anxiety compared to those with low bullying exposure. No significant associations were observed for gender, academic year, residence, or examination failure. After incorporating coping strategies into the multivariate model, maladaptive coping emerged as a strong independent predictor of moderate to severe anxiety (Adjusted OR ≈ 1.18 per unit increase, p < 0.001). Adaptive coping did not show a significant association with anxiety in the adjusted mode. Fig.1 showing conceptual pathway.

## DISCUSSION

The present study investigated the rate of bullying exposure and its connection with depression, anxiety, stress, and coping strategies among undergraduate dental students. Overall, the findings indicate a high burden of bullying and psychological distress, with bullying exposure showing statistically significant associations with all three mental health outcomes, particularly anxiety. Because of the cross-sectional design, correlation does not imply causation; the results of the relationship between bullying, maladaptive coping, and anxiety may also be because of reverse causality (e.g. anxious students might be more prone to report or perceive bullying) or confounding that is not measured.

Striking findings of this study is the high burden of bullying, with nearly three-quarters of students (74.2%) reporting high bullying exposure. This prevalence is higher than that reported in several international or regional studies; for example, a study of dental interns in Karachi found bullying prevalence of 36.8% based on negative acts and 55% by self-labeled experience^7^ suggesting that bullying may be particularly widespread in the local academic context. Traditional power dynamics, competitive academic environments, and limited reporting mechanisms have been identified as contributing factors in dental institutions.^8^

Mental health outcomes revealed concerning patterns. More than half of the participants reported from moderate to extremely severe depression (50.8%), while anxiety levels were particularly alarming, with 54.0% experiencing severe to extremely severe anxiety. These findings are similar to recent meta-analyses indicating that anxiety is the most frequent psychological problem among dental students, often exceeding depression and stress levels.^9^ Similar results have been reported in a study conducted among university students, which documented a high overall prevalence of depression (75%), anxiety (88.4%), and stress (84.4%). In that study, depression levels ranged from normal (25%) to extremely severe (8.6%), however, anxiety was ranging from severe to extremely severe (64.6%), and stress levels were mostly mild to moderate.^10^ On the other hand, most students reported normal stress, suggesting that students may be better able to cope with routine academic demands. In contrast, bullying acts as a long term stressor that significantly contributes to increases anxiety and depression. Similar patterns have been reported distinguishing workload from interpersonal stressors.^11^

The chi-square analyses demonstrated significant associations between bullying exposure and depression, anxiety, and stress, with the strongest association observed for anxiety (χ² = 29.2, p < 0.001). Hence, Students experiencing high bullying were more likely to report severe anxiety, consistent with evidence that bullying triggers anxiety via fear of evaluation, social isolation, and anticipatory stress.^12^

The observed association between bullying and depression aligns with previous studies showing that repeated exposure to negative interpersonal behaviors contributes to feelings of helplessness, reduced self-worth, and emotional exhaustion among dental and medical students.^13^ The stress findings further support the cumulative psychological burden of bullying, particularly when exposure is persistent and unaddressed.

Past studies have highlighted hostel residence as a possible contributor for peer victimization and psychological distress among medical and dental students.^14^ Only a small percentage reported a prior mental health diagnosis, suggesting that the high levels of depression and anxiety observed may be situational and academic-environment driven rather than preexisting, aligning with earlier findings in health professions education.^15^ Furthermore, a study at Aga Khan University reported high stress, anxiety, and depression among undergraduate medical, nursing, and dental hygiene students, with no significant differences across academic years, suggesting that psychological distress persists throughout training despite varying stressors.^16^ Even though finding of this study demographic factors such as gender, residence, and history of exam failure were evaluated, no notable associations with mental health outcomes were observed, except for academic year, which was markedly associated with anxiety levels (χ² = 41.3, p < 0.001). This finding suggests that anxiety may vary across different stages of dental training, third and fourth year students spend more time in clinical environments with direct exposure to clinical supervisors, a bullying hotspot in dental education. Clinical rotations are hierarchical and reliant on supervisor evaluations to advance through the hierarchy, which leads to the development of a power imbalance that can be used to mistreat the students. Clinical year students also encounter other stressors such as patient care duties, anxiety about performance during the process, and anxiety about making errors in the presence of the supervising faculty, while other demographic factors appear less influential in this cohort.

Analysis of the Brief COPE inventory revealed that adaptive coping strategies, particularly active coping and planning were mostly used by students. Though, maladaptive approaches such as self-blame and denial were also prevalent. This mixed coping profile mirrors findings from recent studies suggesting that health professional students often employ adaptive strategies while simultaneously engaging in maladaptive behaviors under high psychological strain.^17^ Spearman’s correlation analysis provided further insight into these dynamics. Bullying exposure showed a moderate positive correlation with maladaptive coping (ρ = 0.403, p < 0.001) and anxiety severity (ρ = 0.427, p < 0.001). Importantly, maladaptive coping demonstrated a strong correlation with anxiety (ρ = 0.648, p < 0.001), indicating that maladaptive coping may act as a key mediating pathway through which bullying influences anxiety outcomes.

Likewise mediation patterns have been reported in recent mental health research among university students.^18^ Interestingly, adaptive coping was also weakly positively correlated with anxiety, similarly reflecting increased coping efforts in response to distress rather than effectiveness, a phenomenon known as “reactive coping escalation” in highly stressed students.^19^ Previous literature on coping with psychological distress reports that digital interventions can have a positive impact on students’ mental health, but the effectiveness varies from person to person depending on how the intervention is used and the specific intervention framework.^20^ These findings support a conceptual pathway which shows exposure to bullying is associated with coping responses, which in turn are linked to psychological outcomes, especially anxiety (Fig.1). This study provides novel evidence on the association between bullying exposure and quantitatively measured anxiety severity (OR ≈ 6.0) among Pakistani dental undergraduates, with maladaptive coping emerging as a potential mediating factor(OR ≈ 1.18). Unlike previous local studies that primarily reported the prevalence of bullying, the current research demonstrates a dose response relationship between bullying severity and psychological distress. Notably, 44.6% of students in the high-bullying group reported extremely severe anxiety, compared to 15.4% in the low-bullying group, highlighting a substantial gradient of risk. These findings suggest that dental institutions should consider routine screening for anxiety among students reporting frequent bullying, as the likelihood of moderate to severe anxiety is significantly higher in this group. From an educational and policy perspective, bullying exposure may be considered an important risk marker for adverse mental health outcomes, warranting its inclusion in student support and monitoring systems. Furthermore, the strong positive association between maladaptive coping and anxiety (ρ=0.648) suggests that coping strategies play a meaningful role in students’ psychological responses. This underscores the potential value of integrating coping-focused psychological support alongside institutional anti-bullying measures.

### Limitations:

The cross-sectional design does not allow causal inference and the single-center setting does not allow generalizability. Students who were either highly or minimally bullied might have been more inclined to respond, which is a source of selection bias.

## CONCLUSION

The study provides local evidence of bullying and its psychological consequences in Pakistani dental students. Higher bullying exposure was linked to severe distress and greater use of maladaptive coping, emphasizing the harm of hostile learning environments. The strong association with anxiety highlights the need for institutional bullying prevention, confidential reporting, early psychological support, and initiatives such as resilience training, peer support, and faculty sensitization to protect student well-being. Anti-bullying policies at dental colleges should be comprehensive, there should be a reporting system that is confidential and accessible, faculty sensitization to adhere to respectful supervision should be a regular part of the curriculum, and the mental health support program should be a part of the core curriculum. Additional longitudinal studies in the future are required to build up time based associations and intervention efficacy.

### AI:

AI-assisted tools (ChatGPT, OpenAI) were used only for grammar and spelling checking. Authors take full responsibility for the content.

### Author’s Contribution:

**PAB:** Conceived and designed the study, analyzed data, drafted the manuscript, and is responsible for its integrity.

**JS:** Supervised the study and critically reviewed the manuscript.

**SR and YAS:** Contributed to data interpretation and manuscript revision.

All authors have approved the final version.
